# Varicella Outbreak Among Recent Arrivals to New York City, 2022–2024

**DOI:** 10.15585/mmwr.mm7321a1

**Published:** 2024-05-30

**Authors:** Krishika A. Graham, Robert J. Arciuolo, Olivia Matalka, Beth M. Isaac, Antonine Jean, Noora Majid, Leah Seifu, John Croft, Bindy Crouch, Michelle Macaraig, Allison Lemkin, Guajira Thomas Caceres, Ramona Lall, Cheryl Lawrence, Erica Silverman, Fabienne Laraque, Alyssa Bouscaren, Jennifer B. Rosen

**Affiliations:** ^1^New York City Department of Health and Mental Hygiene, New York, New York; ^2^Epidemic Intelligence Service, CDC; ^3^New York City Health and Hospitals, New York, New York; ^4^New York City Department of Homeless Services, New York, New York.

**Summary**

**What is already known about this topic?**

In October 2022, the New York City Department of Health and Mental Hygiene (DOHMH) identified a varicella outbreak among persons who recently migrated from or through Central and South America and lived in New York City (NYC) shelters or residential facilities; the outbreak is ongoing.

**What is added by this report?**

The majority of varicella cases (53%) occurred in persons aged 4–18 years, and most (92%) occurred in persons with no documentation of varicella vaccination. The most common sources of transmission included NYC shelters or residential facilities (41.3%) and importation or possible importation (39.4%). School transmission accounted for only 1.2% of cases. Approximately 27,000 varicella-containing vaccine doses have been administered to recently arrived migrant children, adolescents, and adults by vaccination vendors deployed by DOHMH and NYC’s public hospital system.

**What are the implications for public health practice?**

This outbreak highlights the importance of limiting transmission by achieving and sustaining high varicella vaccination coverage and the need for rapid, large-scale vaccination efforts in light of ongoing importations and exposures in U.S. shelters and residential facilities.

## Abstract

Varicella is an illness characterized by a generalized, pruritic rash and transmitted through airborne, droplet, and contact transmission. Although varicella causes mild-to-moderate symptoms in most persons, serious complications, including pneumonia and death, can occur. In October 2022, the New York City (NYC) Department of Health and Mental Hygiene (DOHMH) identified a varicella outbreak primarily involving persons who recently migrated from or through Central and South America and lived in an NYC shelter or residential facility; the outbreak is ongoing. Persons with suspected varicella were reported to DOHMH by city-run shelters and residential facilities, schools, and health care facilities. DOHMH investigations included patient interview and review of medical and immunization records. As of March 8, 2024, a total of 873 outbreak-associated varicella cases were reported. An outbreak-associated case was defined as a clinically compatible rash and either provider diagnosis of or known exposure to varicella in a person who recently had migrated from or through Central or South America or had an epidemiologic link to someone who did. The majority of cases (53.0%) were among children and adolescents aged 4–18 years, and most patients (91.9%) had no documentation of varicella vaccination at the time of symptom onset. In total, 28 varicella-associated hospitalizations and no deaths to date were reported. Among 780 (89.3%) cases with a known source of transmission, the most common sources included shelters and residential facilities (41.3%) and importation or possible importation (39.4%). School transmission accounted for only 1.2% of cases. Ongoing control measures include isolation of infectious persons, quarantine of nonimmune contacts, recommended temporary closure of shelters and residential facilities with evidence of residence-based transmission, and providing or supporting varicella vaccination operations. Approximately 27,000 varicella-containing vaccine doses have been administered to recently arrived migrant children, adolescents, and adults by vaccination vendors deployed by DOHMH and NYC’s public hospital system. This outbreak highlights the importance of limiting transmission by achieving and maintaining high varicella vaccination coverage and the need for rapid, large-scale vaccination efforts given ongoing importations and exposures in shelters and residential facilities.

## Investigation and Findings

### Identification of Varicella Outbreak

Varicella is an illness characterized by a generalized, pruritic rash and transmitted through airborne, droplet, and contact transmission. Although varicella causes mild-to-moderate symptoms in most people, serious complications, including pneumonia and death, can occur. Varicella vaccine is highly effective at preventing infection. Since Spring 2022, New York City (NYC) has welcomed and provided assistance, including health services, legal services, education, and housing to approximately 180,000 migrants, many of whom are seeking asylum in the United States. Approximately 65,000 asylum seekers are currently living in city-run shelters and residential facilities. Many migrants are from countries that do not include varicella vaccine in their routine immunization programs or those where routine immunization programs have been disrupted (*1*). In October 2022, NYC’s Department of Health and Mental Hygiene (DOHMH) identified three cases of varicella among persons living in a residential facility who had recently migrated from or through Central or South America, prompting further investigation. Since identification of those initial cases, an outbreak of varicella has been ongoing among this population.

### Identification and Classification of Outbreak-Associated Varicella Cases

Before 2024, individual cases of varicella in NYC were not reportable by providers; however, reporting of outbreaks (defined as three or more cases) is mandated by NYC Health Code Section 11.03(c) (*2*). Since identification of this varicella outbreak, DOHMH issued provider alerts to emergency departments, hospitals, and federally qualified health centers describing the outbreak and requesting reporting of outbreak-associated cases (*3*). An outbreak-associated case was defined as a clinically compatible varicella rash (i.e., generalized maculopapular and vesicular rash) and either provider diagnosis of or known exposure to varicella or herpes zoster in a person who recently migrated from or through Central or South America since June 2022 or had an epidemiologic link to someone who did (e.g., by school, residence, or migration from other countries to the United States through the southern border). Cases were reported to DOHMH by medical providers, shelters and residential facilities, and schools, with additional case finding through patient interviews, electronic laboratory reports, and syndromic surveillance of emergency department chief complaints and discharge diagnoses *(International Statistical Classification of Diseases, Tenth Revision* codes) indicating varicella. Case investigations included patient interviews, review of medical and immunization records, and identification of venues attended during the incubation period (10–21 days before rash onset) or infectious period (from 2 days before rash onset until all lesions have crusted and no new lesions have appeared for 24 hours). This activity was reviewed by CDC, deemed not research, and was conducted consistent with applicable federal law and CDC policy.*

### Characteristics of Outbreak-Associated Varicella Cases

As of March 8, 2024, a total of 873 outbreak-associated varicella cases was identified, with onset dates during September 12, 2022–March 6, 2024 (Figure). The median patient age was 11 years (range = 2 weeks–70 years); 17.5% of cases occurred among children aged <4 years, 53.0% among children and adolescents aged 4–18 years, and 29.4% among adults aged >18 years (Table). Most (802; 91.9%) patients had no documentation of receipt of varicella vaccine at the time of symptom onset. Overall, 28 varicella-associated hospitalizations have been reported. The median age of hospitalized patients was 22 years (range = 2 weeks–43 years); 15 patients were admitted for complications, including encephalitis, pneumonia, bacteremia, and secondary bacterial skin superinfection, three for a diagnostic evaluation, and 10 for isolation or observation. Nine patients were pregnant at time of infection, five of whom delivered a newborn in NYC with a normal birth exam; four of these pregnant patients were treated with acyclovir. Two patients delivered outside NYC, and two others have not yet delivered. No varicella-associated deaths have been reported.

**FIGURE:**
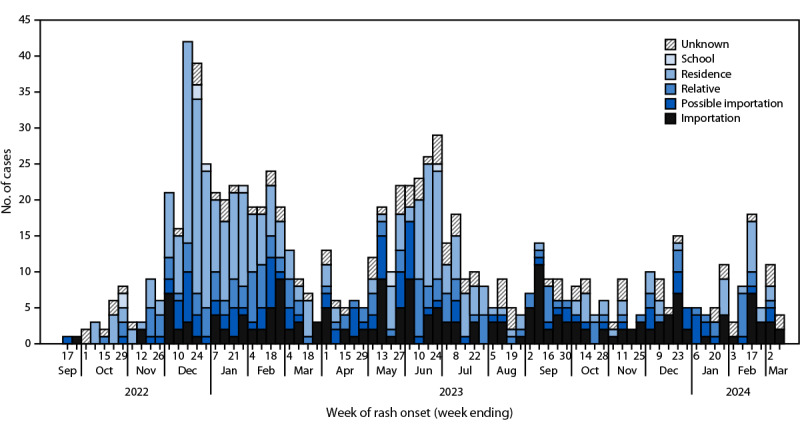
Varicella cases, by week of rash onset and transmission source* — New York City, September 12, 2022–March 6, 2024 **Abbreviation**: NYC = New York City. * Transmission sources are defined as follows: school = patient attended the same school as another patient whose infectious period overlapped with their incubation period, lived in NYC for their full incubation period, and had no known household exposure during their incubation period; residence = patient lived at the same residential facility as another patient whose infectious period overlapped with their incubation period, lived in NYC for their full incubation period, and had no known household exposure; relative = infection likely acquired from a household member whose infectious period overlapped with patient’s incubation period; possible importation = patient arrived in NYC during their incubation period (10–21 days before rash onset); and importation = patient’s entire incubation period (10–21 days before rash onset) occurred while living outside of NYC.

**TABLE:** Characteristics of outbreak-associated varicella cases (N = 873) — New York City, September 12, 2022–March 6, 2024

Characteristic	No. (%)
**Patient age group, yrs (n = 873)**	
<4	153 (17.5)
4–18	463 (53.0)
>18	257 (29.4)
**No. of documented varicella vaccine doses received at the time of symptom onset (n = 873)**	
0	802 (91.9)
1	59 (6.8)
2	12 (1.4)
**Place of residence (n = 873)**	
Shelter or residential facility	820 (93.9)
Private residence	53 (6.1)
**Hospitalizations (n = 28)**	
Complications	15 (53.6)
Isolation or observation	10 (35.7)
Diagnostic evaluation	3 (10.7)
**Pregnant at time of infection (n = 9)**	
Delivery in NYC of newborn with normal exam at birth	5 (55.6)
Delivery outside NYC	2 (22.2)
Not yet delivered	2 (22.2)
Known source of transmission* (n = 780)	
Residence	322 (41.3)
Importation or possible importation	307 (39.4)
Relative	142 (18.2)
School	9 (1.2)

**Abbreviation**: NYC = New York City.

* Transmission sources are defined as follows: residence = patient lived at the same residential facility as another patient whose infectious period overlapped with their incubation period, lived in NYC for their full incubation period, and had no known household exposure; importation or possible importation = all or part of the patient’s incubation period occurred before arrival in NYC (10–21 days before rash onset); relative = infection likely acquired from a household member whose infectious period overlapped with patient's incubation period; school** **= patient attended the same school as another patient whose infectious period overlapped with their incubation period, lived in NYC for their full incubation period, and had no known household exposure during their incubation period.

### Sources of Transmission

Among 780 (89.3%) cases with a known source of transmission, 41.3% of persons were exposed in a shelter or residential facility, 39.4% of cases were importations or possible importations (i.e., all or part of the patient’s incubation period occurred before arrival in NYC), and 18.2% were infected by a household or family member. School transmission accounted for 1.2% of cases.

Patients lived in 105 shelters and residential facilities; a median of three cases occurred in each facility (range = 1–197 cases). Notably, one large residential facility, with approximately 950 rooms used for families, has reported nearly one quarter (197, 22.6%) of all cases. This high percentage was attributed to extensive transmission at that site accounting for most of the cases during the first peak of the outbreak (December 2022–February 2023). The outbreak within this residential facility ended after an extended varicella vaccination campaign, after which the percentage of children with documentation of varicella-containing vaccine or other evidence of immunity increased from 28% in December 2022 to >80% in February 2023. A residential facility outbreak was considered to have ended when no additional cases were reported for two incubation periods (a total of 42 days) after the last case. Importation of cases into NYC is ongoing, with subsequent household spread and transmission across multiple residential facilities coinciding with the opening of new residential facilities.

## Public Health Response

### Isolation, Quarantine, and Post-Exposure Prophylaxis

DOHMH worked closely with NYC agencies that oversee shelters and residential facilities to implement rapid case reporting and isolation and quarantine of susceptible contacts (children and adolescents without documentation of varicella vaccination and adults who report not having had varicella disease) as indicated. Pregnant persons exposed to varicella were screened for evidence of immunity through ascertainment of varicella vaccination records, varicella immunoglobulin G (IgG) results from previous prenatal care records, or through referral for serologic IgG testing. Pregnant contacts without evidence of varicella immunity were referred for postexposure prophylaxis with varicella zoster immune globulin (VariZIG). Beginning in February 2023, DOHMH also recommended temporary closures of sites with evidence of residence-based transmission to new residents.

### Vaccination in Shelters and Residential Facilities and Linkage to Primary Care

During the outbreak, DOHMH and other NYC agencies provided or supported vaccination operations across multiple residential facilities, prioritizing children and adolescents without documentation of varicella vaccination, by deploying vaccination vendors for onsite administration of all routine childhood vaccines. To rapidly facilitate varicella vaccination at residential facilities with multiple varicella cases, varicella vaccination was offered along with measles, mumps, and rubella (MMR) vaccine, to avoid the need to delay MMR for 28 days, because of the 28-day minimum interval recommended between administration of live viral vaccines. Influenza and COVID-19 vaccination and all routine pediatric immunizations required to attend school in NYC were also provided. Adults without documentation of varicella vaccine who reported not having had varicella disease were also offered varicella and MMR vaccination. Approximately 27,000 varicella-containing vaccine doses have been administered to recently arrived migrant children, adolescents, and adults, by vaccination vendors deployed by DOHMH (>2,900 doses) and NYC’s public hospital system, NYC Health + Hospitals (>24,000 doses). Other efforts to increase vaccination included implementing door-to-door education and outreach at shelters and residential facilities to review vaccination services and school immunization requirements and creating linkages to community health centers for primary care and immunization services to ensure that remaining routine immunizations and doses needed to complete vaccination series were given. These efforts include scheduling primary care appointments for children and adolescents and providing technical support for vaccine management (assisting with vaccine ordering and reviewing vaccine storage and handling), depending on availability of clinical services onsite. DOHMH also worked closely with approximately 130 schools to notify families of children and adolescents exposed in school about reported school exposures and to recommend exclusion of susceptible children and adolescents from school until they received varicella vaccination.

## Discussion

This outbreak is ongoing as of March 8, 2024. Most cases (70.6%) have occurred among children and adolescents; however, a substantial number of cases occurred among adults aged >18 years. Many recent migrants in NYC arrived from countries that do not have a routine varicella vaccination program and have a high incidence of varicella (*1*,*4*). In countries that do include varicella vaccination in routine immunization schedules, vaccination programs might have been limited or disrupted because of multiple factors, including the COVID-19 pandemic and political instability (*5*,*6*). In addition, countries of origin were primarily tropical countries where varicella susceptibility among adults is higher; limited published data indicate a lower varicella seroprevalence among young adults than that reported in the United States (*4*,*7*,*8*). Moreover, many persons who recently migrated to NYC currently live in residential facilities. Although many of these facilities have private rooms, some are actual congregate settings, and substantial varicella transmission has been reported in one residential facility with private rooms.

This outbreak highlights the importance of high varicella vaccination coverage and the need for infrastructure to support rapid, large-scale vaccination efforts for persons who recently arrived in the United States from countries not routinely providing this vaccine. At the large NYC residential facility that experienced substantial transmission, further transmission subsided after the percentage of children with varicella immunity (i.e., vaccination or other evidence of immunity) exceeded 80%. Despite multiple exposures in schools, and approximately two thirds of cases occurring in school-aged children, minimal transmission (1.2%) was reported in this setting. This finding is likely attributable to high varicella vaccination coverage among school-aged children because of New York State law requiring documentation of 2 doses of varicella vaccine to attend school grades K–12 (*9*). In NYC, varicella vaccine coverage among kindergarten children during the 2021–22 school year was 96.7% (*10*).

### Implications for Public Health Practice

Ongoing importation of varicella into NYC highlights the importance of migrants having access to varicella vaccine and other vaccines throughout their journey. Efforts to provide varicella and other routine immunizations are continuing in NYC, including through provision of onsite vaccination at residential facilities and navigation of families to primary health care services. City agencies have also set up an arrival center that offers varicella vaccinations to persons who have recently migrated at the time of their arrival in NYC. Exploring strategies to improve migrants' access to varicella and other vaccines early in their migration pathway could help increase varicella immunity in this population and limit introduction of varicella and subsequent transmission in NYC and other U.S. jurisdictions.

Syndromic surveillance and electronic laboratory reporting continue to supplement outbreak case ascertainment. In jurisdictions where individual cases of varicella are not reportable, syndromic surveillance and electronically reported laboratory results might be helpful case finding tools that could aid in identifying and responding to varicella outbreaks.

## Footnotes

* 45 C.F.R. part 46.102(l)(2), 21 C.F.R. part 56; 42 U.S.C. Sect. 241(d); 5 U.S.C. Sect. 552a; 44 U.S.C. Sect. 3501 et seq.
